# Translation and validation of the Serbian primary biliary cholangitis-40 questionnaire

**DOI:** 10.1371/journal.pone.0175697

**Published:** 2017-04-13

**Authors:** Simon Zec, Dusan Popovic, Vera Matovic, Vladimir Nikolic, Ksenija Bojovic, Jasna Jovic, Ljiljana Markovic Denic, Tomica Milosavljevic, Tamara Alempijevic

**Affiliations:** 1 Department of Internal Medicine, School of Medicine, University of Belgrade, Belgrade, Serbia; 2 Clinic for Gastroenterology and Hepatology, Clinical Center of Serbia, Belgrade, Serbia; 3 Department of Gastroenterology and Hepatology, Clinic for Tropical and Infectious Diseases, Clinical Center of Serbia, Belgrade, Serbia; 4 Department of Gastroenterology and Hepatology, Military Medical Academy, Belgrade, Serbia; 5 Institute of Epidemiology, School of Medicine, University of Belgrade, Belgrade, Serbia; Laval University, CANADA

## Abstract

**Background and aims:**

To translate into Serbian and validate the Primary Biliary Cholangitis–40 (PBC-40) and PBC-27 questionnaires.

**Materials and methods:**

Ninety-four consecutive outpatients with the diagnosis of PBC from three departments across two tertiary care institutions in Belgrade were enrolled from February to October 2016. Standard methodology for cultural adaption of healthcare related quality of life questionnaires was used, and included: a forward translation, backward translation and a pilot test of the Serbian PBC-40 on five patients who gave suggestions and comments. For evaluation of the questionnaires, acceptance was shown by the proportion of missing items and the internal consistency was assessed using Cronbach’s α coefficient. The PBC-40 was self-administered under the supervision of an experienced hepatologist. The PBC-27 is a shorter version of the PBC-40.

**Results:**

A total of 92 (97.9%) of the patients were females. The mean age was 59.26 ± 1.05 years and the average length of disease was 60.45 ± 48.314 months. The average PBC-40 score was 85.62 ± 30.46. The total time needed to complete the questionnaire ranged from 7 to 16 minutes. The proportion of missing items was 5.45% (205/3760). Cronbach’s α for the entire scale was 0.93. Reliability for all of the domains of the PBC-40 was above 0.70, except for the domain “Symptoms” (α = 0.52). Overall reliability of the PBC-27 was α = 0.90. Domains “Dryness”, “Symptoms” and “Fatigue” demonstrated reliability below α = 0.70.

**Conclusions:**

The Serbian PBC-40 is a valid and reasonably adequate for use in Serbian PBC patients. The PBC-40 is preferred over the PBC-27.

## Introduction

Health-related quality of life (HRQOL), is an important concept encompassing many aspects of the impact of medical care from the patient’s perspective [1].

Primary biliary cholangitis (PBC) is a chronic, potentially life-threatening, autoimmune cholestatic liver disease exemplified by the presence of autoantibodies: anti-mitochondrial antibodies (AMA), and specific anti-nuclear antibody (ANA) subtypes [1, 2]. Characterized by a loss of immune tolerance, resulting in an immune-mediated destruction of biliary epithelial cells, with subsequent loss of small and medium sized bile ducts, it ultimately leads to cholestasis, inflammation, fibrosis and cirrhosis if left untreated [3]. In keeping with its autoimmune origin, PBC primarily effects women and is associated with a significantly higher symptom burden than other chronic liver diseases (CLD), consequently negatively impacting patient’s HRQOL [3, 4]. Progressing slowly, the most common symptoms are dilapidating fatigue, itch and cognitive impairment which may occur at any point, independent of the histological stage of the disease [4, 5].

Initial investigations assessing cholestatic liver diseases and HRQOL found, that compared to other CLD patients, those with PBC had significantly lower quality of life scores [6]. Further studies investigating the HRQOL of patients with PBC [1, 7, 8], demonstrated an urgent need for a disease specific instrument, separate from other CLD and general quality of life questionnaires including the chronic liver disease questionnaire (CLDQ) [9] and the short form health survey-36 [10]. To meet this necessity, Jacoby et al [11], created the first disease specific quality of life scale for PBC, the PBC-40, which was evaluated and found to have appropriate validity and reliability. The PBC-40 has since been cross-culturally adapted and validated into different languages [12, 13]; alongside the creation of the shorter PBC-27 [12].

As of yet, the only HRQOL instrument adapted for CLD patients in Serbia has been the CLDQ [14]. Consequently, the aim of this pilot study was to translate into Serbian, and validate the PBC-40 and PBC-27 questionnaires, so that these instruments may be used in our PBC patients in future investigations.

## Materials and methods

### PBC-40

The PBC-40 is a self-reported 40-item HRQOL scale, consisting of quality of life statements, divided into six domains related to Fatigue (11 items), Emotional (3 items), Social (10 items), Cognitive (6 items), Itch (3 items), and Symptoms (7 items). Domains including Symptoms, Itch, Fatigue and Cognition refer to the last four weeks, with a 1 to 5-point scale, with 1 corresponding to the minimum “Never”, and 5 labelled as maximum “Always”. The remaining domains, Social and Emotional, do not refer to a specific time, and are also labelled with a 1 to 5-point scale, with 1 representing “Not at all” and 5 “Very much”. The total score is obtained by averaging the 40 items, with a higher score denoting a worse HRQOL. Permission to translate and validate the PBC-40 into Serbian was given by Jacoby et al [11].

### PBC-27

The PBC-27 is a shorter instrument derived from the PBC-40, created in 2010 by Montali et al [12]. Unlike the PBC-40, this scale consists of 27 items divided into seven domains: Symptoms (3 items), Dryness (2 items), Itch (3 items), Fatigue (8 items), Cognitive (5 items), Emotional (3 items) and Social (3 items) [12]. The scoring system is the same as for the PBC-40.

### Serbian version of the PBC-40 –translation and pilot study

Adaption of the PBC-40 was performed according to the accepted methodology for validation of HRQOL questionnaires [15, 16]. A “Forward translation” from the original English into Serbian was followed by a “Backward translation”. Each translation was performed by an independent bilingual translator [14]. At this point, the backward translation was reviewed by Jacoby et al [11], and proven to be adequate and acceptable for use. This Serbian version of the PBC-40 questionnaire was then tested on five PBC patients [14]. A panel of experts consisting of an experienced hepatologist, statistician, and epidemiologist, then convened and these test results were comprehensively discussed, after which, the final version of the Serbian PBC-40 was created [14]; this final version was further tested in 15 patients with PBC.

### Sample and data collection

In this cross-sectional study, 94 consecutive outpatients with the diagnosis of PBC from three departments across two tertiary care institutions in Belgrade, Serbia, were enrolled from February to October 2016. So as to allow adequate time for the initial burden of diagnosis to subside, only patients with duration of disease ≥ 12 months were included. Other inclusion criteria were fluency in spoken and written Serbian language, and age > 18 years. Patients demonstrating any of the following criteria were excluded: illiteracy in the Serbian language, length of disease < 12 months, presence of dementia, or psychosis, co-morbid CLD including: viral hepatitis (hepatitis B virus, hepatitis C virus), alcoholic liver disease, metabolic liver disease (non-alcoholic fatty liver disease, Wilson disease, α1-antitrypsin deficiency, hereditary hemochromatosis), autoimmune liver disease (autoimmune hepatitis, primary sclerosing cholangitis), acute decompensation of CLD and prior liver transplantation [14].

Diagnosis of PBC in the 94 enrolled patients was based on the European Association for Study of the Liver (EASL) guidelines [17], with the presence of any 2 of the 3 following features: biochemical indications of cholestasis on 2 occasions at least 6 months apart; presence of AMA at titres ≥ 1:40; histopathological findings consistent with PBC obtained by transcutaneous liver biopsy [17]. Most patients were diagnosed on the basis of transcutaneous ultrasound guided liver biopsy, performed by an experienced hepatologist. Each biopsy specimen was examined and staged by an expert liver pathologist, in accordance with the histological staging system proposed by Ludwig et al [18]. In specimens demonstrating multiple stages, the most advanced histological features determined the final stage [17]. AMA and ANA Hep2 positivity were also noted. Liver biopsy confirmed the diagnosis of PBC in all patients with negative AMA or ANA Hep2 antibodies.

All patients were outpatients coming for regular biannual check-ups, and the following demographic and clinical data were collected where possible: age, gender, length of disease (months), alanine aminotransferase (ALT), aspartate aminotransferase (AST), γ-glutamyltransferase (GGT), alkaline phosphatase (ALP), total bilirubin, serum albumin levels, international normalized ratio (INR), and prothrombin time (PT). Whether the patient had signs of peripheral edema, or was receiving diuretic therapy was also noted by an attending hepatologist. The Mayo Risk Score was thus calculated, and used as a measure of the severity and impact of disease [19, 20]. All patients gave informed written consent to be enrolled in the study prior to initiation of the PBC-40 questionnaire, which was self-administered under the supervision of an experienced hepatologist.

Our study was approved by the Ethical Committee of the Clinical Center of Serbia (no: 264/48), in keeping with the principles of the Declaration of Helsinki (2000 revision of Edinburgh).

### Statistical analysis

Analytical and descriptive statistics were used. Categorical variables are presented as proportions. Continuous variables are shown as mean ± SD. Pearson correlation was used to identify correlations between the various PBC-40 domains, Mayo Risk Score and length of disease.

The acceptance of the instrument was evaluated by the proportion of missing items [14]. The internal reliability was determined with Cronbach’s α coefficient [21]. An α value of 0.70 was considered internally consistent [22]. Statistical analyses were performed using SPSS 22.0 (SPSS Inc. Chicago, IL, USA).

## Results

Demographic and clinical characteristics of the patients are shown in Table 1. Of the 94 patients included in the study, 92 (97.9%) were females. The mean age was 59.26 ± 1.05 years (range 35–85). From the time of initial diagnosis until the time of enrollment, the average length of disease was 60.45 ± 48.314 months (range 12–246). Markers of cholestasis, mean GGT and ALP values were 62.90 ± 89.57 IU/L and 154.65 ± 92.36 IU/L, respectively. Positive AMA was noted in 86.4% of those tested (n = 88). ANA Hep2 was positive in 54.9% of those tested (n = 82). Liver biopsy was evidenced in 70 patients (74.5%), with the majority (48.6%), showing PBC histopathological stage 1. The mean Mayo Risk Score was 4.34 ± 2.26.

**Table 1:** Demographic and clinical characteristics of the patients (n = 94).

Parameter	Mean ± SD (range)
**Female gender n (%)**	92 (97.9)
**Age (years)**	59.26 ±1.05 (35–85)
**Length of Disease (months)**	60.45 ± 48.31 (12–246)
**ALT IU/L**	30.94 ±23.43 (6–145)
**AST IU/L**	34.77 ± 90.53 (8–215)
**ALP IU/L**	154.65 ± 92.36 (63–508)
**GGT IU/L**	62.90 ± 89.57 (12–692)
**Total Bilirubin μmol/L**	19.10 ± 33.68 (2.7–251.1)
**Albumin g/L**	39.95 ± 6.26 (15.7–68)
**INR**	1.02 ± 0.22 (0.08–2)
**PT (seconds)**	12.88 ± 3.24 (10.2–31.4)
**Mayo Risk Score**	4.34 ± 2.26
**Histopathological stage:**	n (%)
**1**	34 (48.6)
**2**	14 (20)
**3**	8 (11.4)
**4**	14 (20)
**AMA:**	
**Positive**	76 (86.4)
**Negative**	12 (13.6)
**ANA Hep2:**	
**Positive**	45 (54.9)
**Negative**	37 (45.1)

ALT: alanine aminotransferase, AST: aspartate aminotransferase, ALP: alkaline phosphatase, GGT: γ-glutamyltransferase, INR: international normalized ratio, PT: prothrombin time, AMA: anti-mitochondrial antibodies, ANA Hep2: anti-nuclear antibody Hep2

Analysis of the 40 items and the total PBC-40 score is presented in Table 2. The average total score was 85.62 ± 30.46. Regarding missing data, 205 items were left unanswered of the total 3760 (5.45%). Item number 14 had the greatest proportion of missing data, with 9 patients not giving an answer (9.6%). Item 19 demonstrated the least missing data with only one patient not answering (1.1%). From initiation to completion, the total time required ranged from 7 to 16 minutes.

**Table 2:** Analysis and missing proportions of the 40 items of the PBC-40.

Item (Domain)	Description	Mean ± SD	Missing items n (%)
**1 (Symptoms)**	I was able to eat what I liked	2.33 ± 1.09	3 (3.2)
**2**	I ate or drank only a small amount, and still felt bloated	2.57 ± 1.15	4 (4.3)
**3**	I felt unwell when I drank alcohol	0.45 ± 1.00	4 (4.3)
**4**	I had discomfort in my right side	2.50 ± 1.03	5 (5.3)
**5**	I had dry eyes	2.66 ± 1.27	8 (8.5)
**6**	My mouth was very dry	2.86 ± 1.06	6 (6.4)
**7**	I had aches in the long bones of my arms and legs	2.93 ± 1.26	5 (5.3)
**8 (Itch)**	Itching disturbed my sleep	1.55 ± 1.51	5 (5.3)
**9**	I scratched so much I made my skin raw	1.40 ± 1.37	7 (7.4)
**10**	I felt embarrassed because of the itching	1.63 ± 1.61	4 (4.3)
**11 (Fatigue)**	I had to force myself to get out of bed	2.14 ± 1.08	6 (6.4)
**12**	I had to have a sleep during the day	2.70 ± 1.01	6 (6.4)
**13**	Fatigue interfered with my daily routine	2.64 ± 1.17	5 (5.3)
**14**	I felt worn out	2.84 ± 1.04	9 (9.6)
**15**	I felt so tired, I had to force myself to do the things I needed to do	2.29 ± 1.05	5 (5.3)
**16**	I felt so tired, I had to go to bed earlier than usual	2.44 ± 1.00	3 (3.2)
**17**	Fatigue just suddenly hit me	2.87 ± 4.62	4 (4.3)
**18**	PBC drained every ounce of energy out of me	2.11 ± 1.17	8 (8.5)
**19**	Some days it took me a long time to do anything	2.60 ± 1.09	1 (1.1)
**20**	If I was busy one day I needed at least another day to recover	2.37 ± 1.32	4 (4.3)
**21**	I had to pace myself for day-to-day things	2.65 ± 1.27	4 (4.3)
**22 (Cognition)**	I had to make a lot of effort to remember things	2.53 ± 1.30	4 (4.3)
**23**	I had difficulty remembering things from one day to the next	2.21 ± 1.24	7 (7.4)
**24**	My concentration span was short because of PBC	2.18 ± 1.18	7 (7.4)
**25**	I had difficulty keeping up with conversations	1.85 ± 1.19	6 (6.4)
**26**	I found it difficult to concentrate on anything	2.04 ± 1.19	7 (7.4)
**27**	I found it difficult to remember what I wanted to do	2.23 ± 1.08	5 (5.3)
**28 (Social)**	My sex life has been affected by having PBC	2.46 ± 1.24	5 (5.3)
**29**	I feel I neglect my family because of having PBC	1.81 ± 1.64	6 (6.4)
**30**	I feel guilty that I can’t do what I used to do because of having PBC	2.30 ± 1.30	6 (6.4)
**31**	I sometimes feel frustrated that I can’t go out and enjoy myself	1.58 ± 1.19	7 (7.4)
**32**	I tend to keep the fact that I have PBC to myself	2.29 ± 1.47	3 (3.2)
**33**	I can’t plan holidays because of having PBC	3.09 ± 1.24	3 (3.2)
**34**	My social life has almost stopped	2.35 ± 1.16	6 (6.4)
**35**	Everything in my life is affected by PBC	2.44 ± 1.22	4 (4.3)
**36**	PBC has reduced the quality of my life	2.21 ± 1.22	5 (5.3)
**37**	I can still lead a normal life, despite having PBC	1.96 ± 1.02	4 (4.3)
**38 (Emotional)**	Because of PBC, I get more stressed about things than I used to	2.12 ± 1.11	5 (5.3)
**39**	Having PBC gets me down	2.61 ± 1.26	6 (6.4)
**40**	I worry about how my PBC will be in the future	2.24 ± 1.23	3 (3.2)
**Total**		85.62 ± 30.46	205 (5.45)

The reliability of the Serbian PBC-40, as determined using Cronbach’s α coefficient for each individual domain, is shown in Table 3. Only those questionnaires where an entire domain was correctly filled were used in the analysis. Cronbach’s α coefficient for the entire scale was α = 0.93. The highest reliability was seen in the domain “Cognition” (α = 0.95). The domain “Symptoms” had the lowest reliability with an α value of 0.52. All of the other domains demonstrated adequate reliability above α = 0.70.

**Table 3:** Reliability of the Serbian PBC-40 using Cronbach's α coefficient.

Domain	n	Mean ± SD	Cronbach's α Coefficient
**Symptoms**	90	16.24 ± 4.14	0.52
**Itch**	87	4.59 ± 4.09	0.89
**Fatigue**	91	27.47 ±11.33	0.79
**Cognition**	88	13.07 ± 6.36	0.95
**Social**	91	21.43 ± 9.43	0.90
**Emotional**	91	7.88 ± 3.27	0.84

Table 4 shows the reliability of the Serbian PBC-27. The overall reliability of the scale was α = 0.90. The domain “Cognition” had the highest reliability with α = 0.93. The domains “Dryness”, “Symptoms” and “Fatigue” demonstrated inadequate reliability with α coefficients of 0.45, 0.55 and 0.67, respectively. The remaining domains showed adequate reliability.

**Table 4:** Reliability of the Serbian PBC-27 using Cronbach's α coefficient.

Domain	n	Mean ± SD	Cronbach's α Coefficient
**Symptoms**	90	7.99 ± 2.51	0.55
**Dryness**	89	5.48 ± 1.84	0.45
**Itch**	87	4.59 ± 4.09	0.90
**Fatigue**	88	20.48 ± 8.52	0.67
**Cognition**	88	10.83 ± 5.31	0.93
**Emotional**	90	7.79 ± 3.33	0.79
**Social**	90	6.55 ± 3.03	0.86

Table 5 represents the correlations between the domains of the PBC-40, the mean Mayo Risk Score and length of disease. There were no statistically significant correlations between the PBC-40 and the length of disease. The domain “Social” was found to be positively correlated with the Mayo Risk Score, with a Pearson correlation of 0.332. No other domains of the PBC-40 were significantly correlated with the Mayo Risk Score.

**Table 5:** Pearson Correlation between individual domains of the PBC-40, Mayo Risk Score and Length of disease.

Domain	Mayo Risk Score	Length of disease
**Symptoms**	-0.007	-0.003
**Itch**	0.116	-0.176
**Fatigue**	0.156	-0.010
**Cognition**	0.061	-0.034
**Social**	0.332^a^	-0.122
**Emotional**	0.156	-0.201
**Total PBC-40 score**	0.196	-0.091

^a^ Correlation is significant at the 0.01 level (2-tailed).

## Discussion

We herein report the first validation of the PBC-40 and PBC-27, in a Serbian cohort of PBC patients. The aim of this study was to access whether the Serbian versions of the scales could be employed in further HRQOL investigations in our PBC patient population.

Our study sample had similar demographic and clinical characteristics to other PBC cohorts [12, 13], with the exception that only 20% of our cohort had biopsy proven cirrhosis (histological stage 4), less than the number seen in a recent Polish cohort [13].

Our sample had 5.45% missing items. Although seemingly high, similar frequencies have been seen in other HRQOL instrument validations [14, 23, 24]; therefore, acceptance of the Serbian PBC-40 is adequate.

Based on our results, the overall internal consistency of the PBC-40 was outstanding with Cronbach’s α = 0.93, better than that seen in other validations [13]. However, the domain “Symptoms” was found to have a Cronbach’s α coefficient of 0.52, indicating an unsatisfactory level of internal reliability. This result is similar to that of other validations in Italian, Japanese and Polish samples [12, 13]. This domain includes items 1 through 7 and is a heterogeneous domain; items 1–3 address food and alcohol intake, and items 4–7 investigate general discomfort along with eye and mouth dryness. This distinction is important to note, because Montali et al [12], who created and validated the PBC-27, developed a seven domain model by separating items 5 and 6 into a new domain “Dryness”, thus yielding better internal consistency. Low reliability in our population to questions concerning diet was also found in the recently validated Serbian CLDQ [14]. Here the domain “Activity”, which includes two questions related to diet, had inadequate reliability [14], similar to other validations from Germany and Spain [23, 25]. As such, the Serbian version of the CLDQ introduced a new domain titled “Nutrition” to improve reliability [14]. Although the CLDQ is not specific to PBC patients our findings reiterate that, regardless of the underlying etiology of CLD, a unique cultural relationship between diet and disease exists within our population [14]. In the Serbian general population, and especially in those who are disease stricken, diet is of great importance. Namely, patients often associate food intake with a potential cure, often citing that “good health enters through the month”. We therefore believe that our patients tend to tolerate mild physical symptoms more than limitations of diet. In spite of this, we decided not to alter the structure of the Serbian PBC-40. Due to the small number of validations of this instrument, we believe that any alteration would hinder comparisons with other PBC-40 validations.

The overall internal reliability of the Serbian PBC-27 was high (α = 0.90), however, the reliability in domains “Dryness” and “Symptoms” is far lower than that seen by Montali et al [12]. “Fatigue” which was internally consistent in the Italian and Japanese versions of the PBC-27 [12], also demonstrated poor reliability in our study, with an α coefficient of 0.67. Together, these findings illustrate that the PBC-27 is less adequate for use in the Serbian population in comparison to the PBC-40.

The “Social” domain of the PBC-40 was found to have a statistically significant positive correlation with the mean Mayo Risk Score, indicating that patients with a more severe clinical status tend to score higher in this domain. The domain “Social” includes items 28–34, related to how PBC impacts the patient’s sex-life, interpersonal relationships, and ability to travel. We can conclude, based on this correlation, that those patients with more severe PBC, may benefit from a more organized and nurturing social support network around them, thus accounting for this somewhat paradoxical correlation.

## Conclusions

The cross-culturally adapted, and validated Serbian version of the PBC-40 questionnaire was found to be reasonably adequate, and may be used in further HRQOL investigations in our population of PBC patients. The PBC-40 is preferred over the PBC-27.

## References

[1] Rannard A, Buck D, Jones DE, James OF, Jacoby A. Assessing quality of life in primary biliary cirrhosis. Clin Gastroenterol Hepatol. 2004;2(2): 164–74. 15017622 10.1016/s1542-3565(03)00323-9

[2] Purohit T, Cappell MS. Primary biliary cirrhosis: Pathophysiology, clinical presentation and therapy. World J Hepatol. 2015;7: 926–941. doi: 10.4254/wjh.v7.i7.926 25954476 10.4254/wjh.v7.i7.926 PMC4419097

[3] Corrigan M, Hirschfield GM. Aspects of the Pathophysiology of Primary Biliary Cirrhosis. Dig Dis. 2015; 33 Suppl 2: 102–8. 26641487 10.1159/000440755

[4] Mells GF, Pells G, Newton JL, Bathgate AJ, Burroughs AK, Heneghan MA, et al Impact of primary biliary cirrhosis on perceived quality of life: the UK-PBC national study. Hepatology. 2013;58(1): 273–83. doi: 10.1002/hep.26365 23471852 10.1002/hep.26365

[5] Jopson L, Jones DE. Fatigue in Primary Biliary Cirrhosis: Prevalence, Pathogenesis and Management. Dig Dis. 2015;33 Suppl 2: 109–14. 26641884 10.1159/000440757

[6] Younossi ZM, Kiwi ML, Boparai N, Price LL, Guyatt G. Cholestatic liver diseases and health-related quality of life. Am J Gastroenterol. 2000;95(2): 497–502. doi: 10.1111/j.1572-0241.2000.01774.x 10685757 10.1111/j.1572-0241.2000.01774.x

[7] Poupon RE, Chrétien Y, Chazouillères O, Poupon R, Chwalow J. Quality of life in patients with primary biliary cirrhosis. Hepatology. 2004;40(2): 489–94. doi: 10.1002/hep.20276 15368455 10.1002/hep.20276

[8] Jones DE, Bhala N, Burt J, Goldblatt J, Prince M, Newton JL. Four year follow up of fatigue in a geographically defined primary biliary cirrhosis patient cohort. Gut. 2006;55(4): 536–41. doi: 10.1136/gut.2005.080317 16299032 10.1136/gut.2005.080317 PMC1856154

[9] Younossi ZM, Guyatt G, Kiwi M, Boparai N, King D. Development of a disease specific questionnaire to measure health related quality of life in patients with chronic liver disease. Gut. 1999;45(2): 295–300. 10403745 10.1136/gut.45.2.295 PMC1727607

[10] Ware JE Jr, Sherbourne CD. The MOS 36-item short-form health survey (SF-36). I. Conceptual framework and item selection. Med Care. 1992;30(6): 473–83. 1593914

[11] Jacoby A, Rannard A, Buck D, Bhala N, Newton JL, James OF, et al Development, validation, and evaluation of the PBC-40, a disease specific health related quality of life measure for primary biliary cirrhosis. Gut. 2005;54(11): 1622–9. doi: 10.1136/gut.2005.065862 15961522 10.1136/gut.2005.065862 PMC1774759

[12] Montali L, Tanaka A, Riva P, Takahashi H, Cocchi C, Ueno Y, et al A short version of a HRQoL questionnaire for Italian and Japanese patients with Primary Biliary Cirrhosis. Dig Liver Dis. 2010;42(10): 718–23. doi: 10.1016/j.dld.2010.01.004 20163995 10.1016/j.dld.2010.01.004

[13] Raszeja-Wyszomirska J, Wunsch E, Krawczyk M, Rigopoulou EI, Kostrzewa K, Norman GL, et al Assessment of health related quality of life in polish patients with primary biliary cirrhosis. Clin Res Hepatol Gastroenterol. 2016;40(4): 471–9. doi: 10.1016/j.clinre.2015.10.006 26621536 10.1016/j.clinre.2015.10.006

[14] Popovic DD, Kovacevic NV, Kisic Tepavcevic DB, Trajkovic GZ, Alempijevic TM, Spuran MM, et al Validation of the chronic liver disease questionnaire in Serbian patients. World J Gastroenterol. 2013;19(30): 4950–4957. doi: 10.3748/wjg.v19.i30.4950 23946600 10.3748/wjg.v19.i30.4950 PMC3740425

[15] Acquadro C, Conway K, Hareendran A, Aaronson N. Literature review of methods to translate health-related quality of life questionnaires for use in multinational clinical trials. Value Health. 2008;11: 509–521. doi: 10.1111/j.1524-4733.2007.00292.x 18179659 10.1111/j.1524-4733.2007.00292.x

[16] Wild D, Grove A, Martin M, Eremenco S, McElroy S, Verjee-Lorenz A, et al Principles of Good Practice for the Translation and Cultural Adaptation Process for Patient-Reported Outcomes (PRO) Measures: report of the ISPOR Task Force for Translation and Cultural Adaptation. Value Health. 2005;8: 94–104. doi: 10.1111/j.1524-4733.2005.04054.x 15804318 10.1111/j.1524-4733.2005.04054.x

[17] European Association for the Study of the Liver: EASL clinical practice guidelines: management of cholestatic liver diseases. J Hepatol. 2009;51: 237–267. doi: 10.1016/j.jhep.2009.04.009 19501929 10.1016/j.jhep.2009.04.009

[18] Ludwig J, Dickson ER, McDonald GS. Staging of chronic nonsuppurative destructive cholangitis (syndrome of primary biliary cirrhosis). Virchows Arch A Pathol Anat Histol. 1978;379: 103–112. 150690 10.1007/BF00432479

[19] Dickson ER, Grambsch PM, Fleming TR, Fisher LD, Langworthy A. Prognosis in primary biliary cirrhosis: model for decision making. Hepatology. 1989;10(1): 1–7. 2737595 10.1002/hep.1840100102

[20] Angulo P, Lindor KD, Therneau TM, Jorgensen RA, Malinchoc M, Kamath PS, et al Utilization of the Mayo risk score in patients with primary biliary cirrhosis receiving ursodeoxycholic acid. Liver. 1999;19(2): 115–21. 10220741 10.1111/j.1478-3231.1999.tb00020.x

[21] Cronbach LJ. Coefficient alpha and the internal structure of tests. Psychometrika. 1954;16: 297–334.

[22] Nunnally J, Bernstein JC. Psychometric theory 3rd Ed. New York: McGraw-Hill; 1994.

[23] Häuser W, Schnur M, Steder-Neukamm U, Muthny FA, Grandt D. Validation of the German version of the Chronic Liver Disease Questionnaire. Eur J Gastroenterol Hepatol. 2004;16(6): 599–606. 15167163 10.1097/00042737-200406000-00014

[24] Schulz KH, Kroencke S, Ewers H, Schulz H, Younossi ZM. The factorial structure of the Chronic Liver Disease Questionnaire (CLDQ). Qual Life Res. 2008;17(4): 575–84. doi: 10.1007/s11136-008-9332-7 18389385 10.1007/s11136-008-9332-7

[25] Ferrer M, Córdoba J, Garin O, Olivé G, Flavià M, Vargas V, et al Validity of the Spanish version of the Chronic Liver Disease Questionnaire (CLDQ) as a standard outcome for quality of life assessment. Liver Transpl. 2006;12(1): 95–104. doi: 10.1002/lt.20551 16382456 10.1002/lt.20551

